# A Baseline Quantitative Analysis of Technology-Facilitated Gender-Based Violence Against Women with Disabilities in South Africa

**DOI:** 10.3390/bs16050745

**Published:** 2026-05-11

**Authors:** Lieketseng Ned, Babalwa Tyabashe-Phume, Eunice Tunggal, Karen Soldatić

**Affiliations:** 1 Division of Disability and Rehabilitation Studies, Department of Global Health, Stellenbosch University, Cape Town 7505, South Africa; babalwat@uj.ac.za; 2 Department of Social Work, Faculty of Humanities, University of Johannesburg, Johannesburg 17011, South Africa; 3 Canada Excellence Research Chair in Health Equity and Community Wellbeing, Faculty of Community Services, Toronto Metropolitan University, Toronto, ON M5B 2K3, Canada; eunice.tunggal@torontomu.ca (E.T.); ksoldatic@torontomu.ca (K.S.)

**Keywords:** digital violence, South Africa, gender-based violence, technology, disability, intersectionality

## Abstract

The rapid proliferation of digital technologies has transformed the landscape of gender-based violence globally. This quantitative study used an online survey to explore the experiences of women with disabilities in relation to technology-facilitated gender-based violence (TFGBV) in South Africa. Findings from 204 participants highlight patterns across age, province, education, employment, income, disability type, and forms of TFGBV experienced, and how TFGBV may differ at intersections of these factors. They show that cyberbullying, hacking, and hate speech were the most prevalent forms of TFGBV, disproportionately affecting women with various disabilities. The study further reveals how socio-economic disadvantage, manifested in limited access to secure technologies, digital literacy, and support systems, intensifies exposure to harm and constrains access to justice. The study calls for inclusive, power-conscious approaches to research, policy and interventions that centre lived experiences of women with disabilities. Addressing TFGBV in low- and middle-income countries (LMICs), therefore, requires not only legal reform and digital safety initiatives but also broader strategies for socio-economic empowerment and systemic transformation to end gendered–disability violence in both the material and virtual world.

## 1. Introduction

Described as the first major pandemic of the social media age, the COVID pandemic prompted an even greater reliance on digital technologies, thus affording perpetrators with different avenues to commit conventional and novel forms of gender-based violence (GBV) (Bansal et al., 2023; Dunn, 2020; Henry & Powell, 2015; Plan International, 2020; United Nations Women, 2020). This includes introducing entirely new and distinct forms of harm that were previously unimaginable or offline in nature (Bansal et al., 2023; Barter & Koulu, 2021; Dunn, 2020; Hinson et al., 2018). Following this, many countries reported increasing online abuse and an increased traffic to the government helpline for adults reporting intimate partner abuse was noted (Economist Intelligence Unit, 2021; United Nations Women, 2020). Additionally, more women and girls reported some form of online abuse and violence or online harassment, including cyberstalking and online sexual harassment (United Nations Women, 2015; Plan International, 2020). Notably, these have come to be known as technology-facilitated gender-based violence (TFGBV), also referred to as technology-facilitated violence against women (TFVAW) (United Nations Women, 2022).

While there is limited consensus on what constitutes TFGBV, in this paper, TFGBV encompasses “any act that is committed, assisted, aggravated or amplified by the use of ICTs or other digital tools, that results in or is likely to result in physical, sexual, psychological, social, political or economic harm, or other infringements of rights and freedoms” (United Nations Women & WHO, 2023, p. 3). This includes a range of behaviours perpetrated through everyday technological advances, such as mobile devices, social media platforms, interactive computer games, text messaging, email or any other related technologies. Common tactics used by perpetrators include hacking, doxxing (posting personal and sensitive information including home and work addresses, telephone numbers, email addresses and family names without permission), image-based sexual abuse (any use of imagery, often sexual in nature, to objectify, exploit, humiliate or harass), impersonation, threats, and unwanted messaging and posting (Backe et al., 2018; Dunn, 2020; Henry et al., 2020; Hinson et al., 2018; United Nations Women & WHO, 2023; United Nations Population Fund, 2022). Manifestations of TFGBV may include combinations of tactics perpetrated online or continued offline (Hinson et al., 2018; Posetti et al., 2021).

Global estimates of the prevalence of TFGBV are consistently high, although the numbers vary depending on the specific forms of TFGBV. A recent global study conducted in 45 countries, with a sample of 100 per country, found that 38% of women surveyed had personally experienced online violence (Economist Intelligence Unit, 2021). Similarly, a 2020 survey conducted across 22 countries found that 58% of girls have experienced online abuse, with most girls reporting their first experience of social media harassment between ages 14 and 16 (Plan International, 2020). It is, however, likely that this existing prevalence data underrepresents the true scope of the problem due to widespread underreporting, a common issue across all forms of gender-based violence, as well as the absence of consistent definitions, measurement tools, and other methodological limitations (United Nations Women & WHO, 2023).

Slut-shaming on social media operates as a distinct form of gendered violence, one that is systematic rather than incidental. It refers to the public humiliation of overwhelmingly women and girls for perceived sexual behaviour that violates social norms (Miano & Urone, 2024). On platforms like Facebook, this shaming is structurally embedded. Papp et al. (2015) found evidence of a reverse sexual double standard, where women who report slut-shaming are themselves judged more harshly than the perpetrators, effectively silencing disclosure. The harm is not merely reputational. Urone et al. (2024) identify distinct vulnerability pathways through which slut-shaming shapes self-determination, with young adults who have experienced prior marginalisation, including those with disabilities, facing compounded risk. Psychosocial precursors matter too: Miano and Urone’s (2024) systematic review identifies peer dynamics, gender norms, and digital anonymity as consistent drivers of slut-shaming behaviour among adolescents and young adults. Bean et al. (2024) extend this geographically, showing that slut-shaming on social media is not a Western phenomenon. Instead, Vietnamese youth demonstrate similar normative frameworks that excuse perpetrators and blame targets. Taken together, this body of evidence establishes slut-shaming as a tech-facilitated harm with gendered architecture, scalable reach, and disproportionate consequences for already marginalised women.

The current state of evidence, as reported by United Nations Women and WHO (2023), reveals that TFGBV is still relatively nascent, with certain aspects of TFGBV more studied than others. For example, this report reveals that most research focuses on the forms and prevalence of TFGBV as well as its harmful impacts (primarily because this information is integral to convincing policymakers), with fewer articles studying risk factors and drivers as well as contexts of TFGBV. Regarding forms of violence, many qualitative studies often analyse technology-facilitated sexual violence, harassment, bullying, coercion, stalking and exploitation. In contrast, quantitative studies are often limited to conceptualisations of TFGBV and very few studies compare how different technology-based tactics are used for perpetrating TFGBV. The report also shows that a large share of reviewed articles analyse the gendered impact of different forms of TFGBV (using a range of qualitative and quantitative research methods). Regarding context, social networking sites are the most studied contexts of TFGBV, followed by communication technologies and personal online accounts, with fewer research studies investigating violence on dating or entertainment sites, as well as GPS-based and ‘smart home’ technologies. However, there remains little evidence on potential risk factors or drivers of TFGBV. This may be primarily because studies often lack the sample size, diversity of study participants, and/or collection of variables needed to disaggregate data by intersectional identities and experiences of TFGBV. Moreover, characterisations of groups are at greatest risk of different types of technology-facilitated violence, or comparisons of risk factors across contexts are frequently missing from the existing research base. At the same time, most population-based studies lack an intersectional analysis in the presentation of their findings. For example, while data findings are often disaggregated by age and sex, fewer studies include variables like sexual orientation, gender identity, income, race, ethnicity, dis/ability or migrant status in their analysis.

For people with disabilities, increased digital inclusion offers greater opportunities for self-representation, communication and participation (Hameed et al., 2025). However, this inclusion also exposes them to entrenched forms of discrimination, harmful stereotypes, and subtle forms of ableism, dynamics that, according to emerging evidence, are especially pronounced for women and girls with disabilities (Hameed et al., 2025). However, the limited TFGBV research among people with disabilities presents a significant gap. There are currently no prevalence estimates of TFGBV among people with disabilities in South Africa—a population that comprises 7.7% of the general population. South Africa is already known for its high rates of offline violence. For example, in the first three months of 2023, 6289 people were violently killed (969 of whom were women), 10,512 women were raped, and 15,000 women were violently assaulted in gender-based violence incidents (South African Police Service, 2023). Even these striking statistics are likely an underrepresentation, due to underreporting. In a study of “developed” countries, more than half of women with disabilities had experienced sexual exploitation by the time they reached adulthood (Brownridge, 2006), and small-scale ID studies from South Africa suggest that similar vulnerabilities exist in this context (Dickman & Roux, 2005; Phasha & Nyokangi, 2012). Yet, despite strong evidence of an elevated risk of violence for disabled people internationally and the high national prevalence of violence generally, the intersection between disability and GBV is relatively under-reported. It is thus likely that the same violence is experienced in the digital spaces, primarily because, as emerging international evidence indicates, TFGBV is a by-product of deeply rooted gender norms and systemic inequalities that may intersect with racism, homophobia, transphobia, ableism, and other discriminatory structural dynamics (Barter & Koulu, 2021; Dunn, 2020; Henry et al., 2020; United Nations Women & WHO, 2023). Consequently, women, young people, people with disabilities, sexual, gender, religious and ethnic minorities, First Nations peoples and those with intersecting marginalised identities are more vulnerable to online violence (Afrouz, 2023; Bansal et al., 2023; Dunn, 2020; Henry et al., 2020; Lenhart et al., 2016; Plan International, 2020; United Nations Women, 2022; World Health Oorganization, 2020).

In South Africa, women with disabilities navigate intersecting oppressions shaped by race, class, geography, and access to services. The digital divide further exacerbates their vulnerability, limiting access to secure technologies and safe online environments (Hameed et al., 2025). However, data that exists on TFGBV is not aggregated according to disability, and African data is especially limited. For example, the existing TFGBV studies in the region (Sam et al., 2019; Mooketsi, 2018; Mulisa & Getahun, 2018; Edwards et al., 2018; Internet Sans Frontières, 2019; Idongesit, 2014; Makinde et al., 2016) are not clear if any of them included women and girls with disabilities. Our recent scoping review of literature in LMICs found only two articles from South Africa—which also did not include those with disabilities, though some participants in the latter study were labelled as having “behavioural problems” (Tyabashe-Phume et al., 2025). The first study tested whether digital, interactive chatbots could help young women across South Africa stay safer by changing their attitudes and skills (De Filippo et al., 2023). The second study explored the impact of social networking sites on adolescent females, the risks involved in online dating, and any detrimental effects (Alberts & Kheswa, 2017). Only one disability-focused intervention study was found in South Africa. The study used an eight-week WhatsApp programme to educate participants with intellectual disabilities on safe and responsible digital practices, aiming to mitigate risks of abuse (Marnewick et al., 2022). These study outcomes highlighted the importance of tailored digital literacy programmes for vulnerable populations.

What can be deduced from the review is that extant research primarily emerges from high-income countries (HICs), it usually focuses on certain forms of online violence such as cyberbullying and sexual harassment, and/or does not include people with disabilities (Afrouz, 2023; Backe et al., 2018; Dunn, 2020; Kim & Ferraresso, 2023). In addition, in LMICs, the vast majority of research about the intersection of disability and digital technology has focused on access and accessibility (Ellis & Kent, 2011; Trevisan, 2014), especially in relation to online government platforms for accessing disability specialist service provision (Goggin & Soldatić, 2022). There is little evidence on the various manifestations of TFGBV and its intersections with disability in the context of low- and middle-income countries, such as South Africa.

The concept of intersectionality, introduced by Crenshaw (1989), highlights the complex and overlapping dimensions of identity and oppression, and emphasises that experiences of discrimination cannot be fully understood through single-axis frameworks, as they are shaped by multiple, intersecting systems of power such as race, gender, disability, and heterosexism (McMahon et al., 2025). This framework calls for a rethinking of how identity and discrimination are conceptualised, centring the need to understand how marginalisation operates at both individual and structural levels (Wickenden, 2023; McMahon et al., 2025). Building on this foundation, our research applies an intersectional lens to TFGBV against women with disabilities. It is crucial to acknowledge the role of interpersonal, institutional, and systemic power dynamics in shaping both violence and responses to it (McMahon et al., 2025). Without such a perspective, research risks reproducing dominant structures by ignoring how disability, gender, and other social locations converge to intensify vulnerability. Using intersectionality allows us to capture the layered realities of women with disabilities who often face compounded forms of exclusion online and offline. This paper seeks to uncover patterns of vulnerability and resilience that can inform more inclusive policy, prevention, and response strategies. Intersectionality is defined with emphasis on the impact that these interactions have on power dynamics and relationships (Wickenden, 2023; Bauer et al., 2021). In LMICs, such as South Africa, the intersectionality of disability with other markers of socio-cultural and material inequality radically increases (South African Human Rights Council, 2017). Our study endeavoured to fill this gap in the incomplete understanding of a complex and rapidly growing phenomenon. Specifically, the aim of this paper is to explore how intersecting identities, namely, disability, age, race, geographic location, level of education, and socio-economic status, shape the types and frequency of TFGBV experienced.

## 2. Methods

This paper is part of a broader mixed-methods research study aimed at understanding the nature, scope, and impact of TFGBV against women with disabilities in South Africa. The research comprises three phases, including: (1) a completed scoping review of the literature on TFGBV against women with disabilities in low- and middle-income countries (Hameed et al., 2025; Tyabashe-Phume et al., 2025); (2) a quantitative online survey distributed nationally throughout South Africa, aimed to characterise patterns of TFGBV among women with disabilities; and (3) semi-structured qualitative interviews with survivors of TFGBV with disabilities. This paper focuses on the online survey data.

The study adopted a participatory action research (PAR) approach (Cornish et al., 2023). Conducting participatory action research on GBV for women with disabilities in South Africa required a systematic approach that prioritised their voices and experiences. We conducted the project in a collaborative, inclusive, and empowering manner that promotes disability justice, rights, and transformative change. For this quantitative phase, we co-developed all relevant and accessible data collection tools with one GBV organisation and two user-led organisations of people with disabilities (OPDs), as partners for both framing the study and conducting data collection, analysis and interpretation of findings. This methodological approach of ‘lived expertise’ was critical to the overall study design. These research partners were identified from the conceptualisation of the project and funding application stages. In addition to contributing to the co-design process, these partner organisations also engaged in checking the accessibility of the co-designed survey content, format and accessibility. Each section of the final co-designed survey was extensively tested by a team of researchers with and without disabilities, and partner organisations. This form of co-design was informed by the engaged participatory action research approach and was oriented to prioritise the voices of community stakeholders. Co-design with stakeholders also ensured that the survey would appropriately capture experiences of TFGBV, as well as essential questions to assess current TFGBV reporting and response methods (Cornish et al., 2023; Zelenko et al., 2021).

### 2.1. Study Population and Sample

The population of interest in this study was women with disabilities who are above the age of 18 in South Africa, and who have experienced gender-based violence online. We initially planned to have 200–300 participants for the survey. While, for statistical purposes, we wanted to hear from 5 to 10% of the possible population, this was not possible due to significant issues related to accessing this population group. Issues of digital accessibility and affordability are prominent issues faced by people with disabilities and compound their access to the digital realm. The call for participation in the online survey was sent to all research partner organisations and shared on the most common social media platforms. The partner organisations further distributed the call to participate via their communication lists, newsletters and social media pages. We estimated on this front that these organisations would each have the capacity to draw in around 3–5% of their membership base, premised upon a newsletter dissemination list and membership and/or client reach of a total of around 5000 people with disabilities, with a gendered disability population of approximately 167 to 300 participants. Additionally, researchers also conducted educational information meetings to raise awareness of TFGBV in community settings (including libraries and community events). After these community information sessions, the survey was made available to community members who identified as having experienced TFGBV.

The sample in this online survey comprises 411 responses, with a final sample of 204 responses, once duplicate participants (indicated by participants providing multiple survey responses) and incomplete surveys were excluded.

By including all women (cisgender and trans women with disabilities), we hoped this survey would allow us to establish an estimate of the community prevalence of TFGBV that would enable the results to be rigorously disaggregated across the complexity of gendered–disability lines within the South African context. As part of their dissemination and recruitment roles, partner organisations were asked to share the survey link via their social media pages. Dissemination materials provided all participants with detailed information about the purpose of the study, their rights as participants, and the voluntary nature of their participation.

### 2.2. Data Collection and Analysis

The online survey was hosted on REDCap from January 2025 to May 2025 and was available in five commonly spoken languages in South Africa, including English, IsiXhosa, IsiZulu, Sesotho and Afrikaans. The survey included both closed-ended and open-ended questions, covering experiences of TFGBV, socio-demographic information, and disability-related characteristics. The introductory section of the survey asked users about the perspective from which they are responding to the questions. For example, for someone who is a survivor of violence, or as someone who witnessed and/or reported violence or as a caregiver of a survivor—this option gave us the opportunity to evaluate responses with awareness of potential biases of proxy respondents informing results (Dagne et al., 2025). Subsequently, demographic questions regarding age, gender, and disability status screened participants (women identifying persons with disabilities who were 18 years old and above) to continue to the main survey body, which asked for details about disability type (see Appendix A), as well as forms of violence experienced, platforms through which TFGBV occurred, and responses of survivors.

Participants were asked about their experiences of TFGBV. For each form of TFGBV, there was a combination of a standardised definition (see Appendix B), followed by a series of closed-ended and open-ended questions to capture the key characteristics of the experience. For example, cyberbullying was first defined for respondents as repeated and intentional attempts to harm another person online, including physical, social, or emotional harm, often involving a power imbalance in which the targeted individual feels vulnerable or unable to stop the behaviour. Providing definitions ensured a shared understanding of the construct before participants responded. These definitions of forms of TFGBV can be viewed in Appendix B.

These preceding questions included listing the year or years in which the violence occurred (with the option to leave the item blank if unsure), indicating the frequency of the violence (once, more than once but fewer than five times, more than five times, or unsure), and identifying the context or platform where the violence occurred. For context, respondents could select multiple options from a predefined list of online platforms and communication channels (e.g., email, social media platforms, online games, text or video chat rooms, messaging applications), with an option to specify another platform or indicate uncertainty. Participants were also asked to identify who perpetrated the violence using a multiple-response checklist which included categories such as stranger, friend or peer, family member, employer or supervisor, acquaintance, other (with space to specify), or unsure.

To complement the closed-ended items, the survey concluded with an open-ended prompt inviting participants to describe their experience in their own words, if they wished to do so. This narrative response option allowed respondents to provide additional context, elaborate on the nature of the violence, and share details not captured by the predefined response categories. Together, the combination of closed-ended and open-ended questions enabled both systematic measurement and richer qualitative insight into participants’ experiences of TFGBV.

Data were analysed using SPSS (version 30). Descriptive statistics were first analysed to summarise the sample’s demographic characteristics (e.g., age, gender identity, educational level) and the distribution of key outcome variables related to gender-diverse-based violence. Descriptive analyses, including frequencies, percentages, means, and standard deviations, provide essential context for understanding the composition of the study population and the prevalence of experiences of TFGBV. Cross-tabulations were used to examine associations between demographic variables and experiences of TFGBV.

### 2.3. Ethics

This study was conducted in accordance with ethical standards governing research with human participants, particularly those from vulnerable populations, as required by two research institutions: Stellenbosch University (approval reference: N24/07/091) and Toronto Metropolitan University (approval reference: REB 2024-519), respectively. Completion of the survey in full afforded participants a voucher of 100 South African rands as a token of thanks for their time, as guided by Stellenbosch University’s guidelines on payment of participants. Due to the sensitive nature of the topic and the potential risks of re-traumatisation, the survey was designed to minimise participant distress, which was reviewed through the co-design and piloting with civil society partners. Participants were not required to answer all questions and were able to withdraw at any point without any consequence. To ensure accessibility, the survey was designed using plain language and compatibility with screen readers, and assistance was offered to participants upon request. In addition, the survey was translated into five commonly spoken languages in South Africa, including English, IsiXhosa, IsiZulu, Sesotho and Afrikaans. Informed consent was obtained electronically before participants accessed the full questionnaire. Confidentiality and anonymity were strictly maintained, and no identifying information was collected. Data were securely stored on a password-protected server in compliance with the Protection of Personal Information Act (POPIA) of South Africa (Republic of South Africa, 2013). At the end of the survey, participants were given an option to select whether they required counselling services. For those who needed such support, there was an option on the survey to place their contact details while protecting their anonymity from the research team, yet generating automated access to professionally trained GBV counsellors through one of the partner organisations.

## 3. Results

### 3.1. Participants’ Demographics

Data were collected from 204 participants who ranged in age from 18 to 74 years. The largest proportion fell within the 18–29 age group (59.3%, *n* = 121), followed by those aged 30–39 (31.4%, *n* = 64) and 40–49 (5.9%, *n* = 12). Smaller proportions were found in the 50 and above (3.4%, *n* = 7) age groups. Overall, the sample was predominantly concentrated among younger adults, particularly those in their mid- to late twenties. The majority of participants identified as African/Black (*n* = 138), Coloured^1^ participants constituted 20.1% of the sample, White participants accounted for 8.8%, and a smaller proportion of respondents (3.4%) identified as Indian. Xhosa was the most commonly reported main language, spoken by 48.5% of participants. English was reported by 32.7%, followed by Afrikaans at 9.6%. Sotho and Zulu were each reported by 3.6%, while Tswana accounted for 1.2%. Pedi and Tshivenda were each reported by 0.4%. Sexual orientation included lesbian (49.5%), straight (34.8%) and 11.3% bisexual participants as the largest groups. No participants self-identified as trans women. This may be due to the focus on disability and TFGBV in recruitment flyers and the partner organisations involved. Moreover, the high representation of participants identifying as lesbian—which is an irregular statistic in the South African context—may be attributed to non-binary or transgender identities being misattributed as sexuality rather than gender due to terminological differences (Lynch et al., 2021). Alternatively, this may be due to more lesbian-identifying women also identifying as having a disability; potentially a result of LGBTI+-related physical violence creating conditions of disability (Lake, 2017; Mutero, 2026). However, this association between homophobic violence and the development of disability has yet to be explored in South Africa and is outside the scope of this study.

Most participants resided in the Western Cape, representing 57.8% (*n* = 118) of the sample. The Eastern Cape followed at 25% (*n* = 51). Smaller proportions were from KwaZulu-Natal (*n* = 13), Northern Cape (*n* = 8), Gauteng (*n* = 5), Limpopo and Mpumalanga (each *n* = 3), Free State (*n* = 2), and North West (*n* = 1). The majority of participants (58.8%) lived in townships, with 11.3% living in informal settlements, and the least number of participants 7.4% reported living in rural areas.

#### 3.1.1. Types of Disabilities

Among the 204 participants, multiple disability types were reported, with some individuals experiencing more than one. The most frequently reported disability type was psychosocial disability (31.9%), followed by pain-related (30.9%), mobility/dexterity (25%), hearing (19.6%), and vision (16.2%). Memory-related (13.7%), learning (6.9%), developmental/neurodivergence (6.9%), and other self-identified disabilities (0.5%) were also reported. This distribution highlights a high prevalence of psychosocial, cognitive, and pain-related conditions within the sample, with considerable overlap between disability types.

Across the different residential areas, disability types varied notably, with some patterns emerging. Among township residents, psychosocial disabilities were particularly prevalent (20.1%), followed by memory difficulties (10.3%) and pain-related disabilities (13.2%). Township participants also had the highest numbers for hearing (10.8%) and vision (7.4%) disabilities. In informal settlements, mobility or dexterity disabilities were most common (5.9%), alongside pain-related disabilities (3.4%). Rural areas had fewer cases overall, with vision disabilities (3.9%) being the most reported. In the suburbs, pain-related disabilities were the most frequent (7.4%), followed by psychosocial disabilities (5.9%). Urban participants showed relatively even distribution across disability types, with mobility or dexterity (3.9%) and pain-related disabilities (3.9%) being most common.

#### 3.1.2. Level of Education

The largest group (41.7%) had completed secondary schooling, with some tertiary education (TVET certificate, college diploma, or Bachelor’s degree), followed by those with only a secondary school certificate (27%). Participants who did not complete secondary school accounted for 26%, and those with postgraduate qualifications accounted for 4.9%. One participant (0.4%) reported an alternative form of education.

#### 3.1.3. Socio-Economic Status

Nearly half of respondents (46.1%) reported being unable to work or involved in unpaid vocation, volunteer or unpaid caregiving work. While 43.1% of the participants reported being unemployed. A small number (10.8%) indicated that they were employed. Some participants did not disclose their income; of those who did, 147 participants had an income of less than R5000, while 24 participants earned between R6000 and R20,000, 5 participants had an income above R21,000, and only 2 participants earned more than R31,000. Additionally, two participants were unsure if they received any social assistance money (e.g., disability grant) in the last 12 months, while 41 did not receive the grant, but a majority of the participants (*n* = 161) did receive a social assistance grant.

### 3.2. Prevalence of TFGBV

Participants reported experiencing multiple forms of TFGBV, with cyberbullying being the most prevalent (*n* = 88, 43.1%). This was followed by hate speech (*n* = 51, 25.0%), hacking (*n* = 49, 24.0%), and defamation (*n* = 39, 19.1%). Cybergrooming was reported by 31 participants (15.2%), while threats affected 28 participants (13.7%). Cyberstalking was experienced by 25 participants (12.3%), and exploitation by 19 participants (9.3%). Less common forms included technology restriction and isolation (through coercive or otherwise undue control) (*n* = 16, 7.8%), online impersonation (*n* = 16, 7.8%), doxxing (*n* = 15, 7.4%), image-based sexual abuse (*n* = 12, 5.9%), surveillance and cyberstalking (*n* = 7, 3.4%), and others not specified (*n* = 2, 1%). These findings reveal that while certain forms of TFGBV, such as cyberbullying and hate speech, are widespread, a substantial proportion of participants are subjected to multiple, overlapping types of online violence.

### 3.3. Intersectionality

#### 3.3.1. Disability Type and Type of TFGBV Experienced

**Figure 1:**
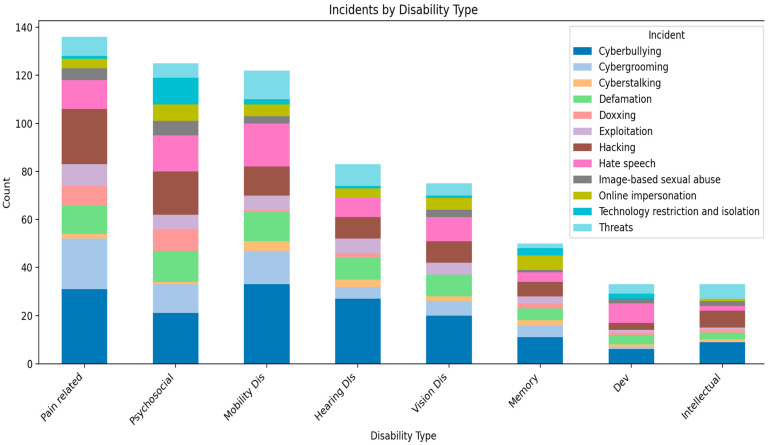
Disability type and TFGBV experienced.

Figure 1 below illustrates the forms of TFGBV experienced by participants per disability type. The most frequently reported type of TFGBV across all disability types was cyberbullying, with the highest proportion among participants with mobility disabilities (*n* = 33) and pain-related disabilities (*n* = 31). This was followed by hacking, particularly among participants with pain-related disabilities (*n* = 23) and psychosocial disabilities (*n* = 18). Hate speech was also prevalent, most notably among participants with mobility disabilities (*n* = 18) and psychosocial disabilities (*n* = 15). Cybergrooming was reported most frequently among those with pain-related disabilities (*n* = 21), while threats were particularly common among mobility disabilities (*n* = 12) and hearing disabilities (*n* = 9). The least frequently reported forms of TFGBV were technology restriction and isolation, with the highest proportion among psychosocial disabilities (*n* = 11) and memory disabilities (*n* = 3), and image-based sexual abuse, which was highest among psychosocial disabilities (*n* = 6).

The mean number of cases per TFGBV type across disability groups ranged from 2.38 (technology restriction) to 22.25 (cyberbullying). The widest range in case counts across disability types was observed for cybergrooming (range = 21), followed by hacking (range = 20), while technology restriction had the smallest range (range = 11).

#### 3.3.2. Race and Type of TFGBV Experienced

Across all racial groups, cyberbullying was the most prevalent incident, followed by hate speech and hacking. TFGBV incidences are concentrated among African/Black respondents (65.5%), followed by Coloured respondents (24.8%), then White (7.55%) and Indian respondents (2.16%). African/Black respondents account for the largest percentage (63.64%) of cyberbullying, followed by Coloured respondents (29.55%), White respondents (4.55%), and Indian respondents (2.27%) experiencing the least percentage of cyberbullying. African/Black respondents had stacked shares throughout all incidents, often exceeding half of all cases in those incident types. In contrast, other racial groups contribute smaller fractions; their category means and medians are correspondingly lower, and their modes sit at the minimum values observed across the distribution. These are highlighted in Table 1.

Central tendency across race groups highlights this concentration—where the overall incident medians for cyberbullying and hacking sit in the mid-to-high single digits within categories, African/Black group medians are above those benchmarks, while some other groups cluster near the lower quartiles. Modes for several incidents are close to 1 in smaller groups (indicating many low-count cells), whereas higher modes in African/Black categories reflect a greater mid-distribution of cases rather than sparse, isolated spikes. Range is also widest for categories with larger totals, indicating both more frequent and more varied exposure.

The incident profile by race displays three concurrent features: (a) concentration—majority of incidents borne by African/Black respondents, (b) breadth—elevated exposure across multiple incident types, not just cyberbullying, and (c) dispersion—wider ranges and higher means within high-burden categories. The correlation structure suggests that once exposure is elevated in a race group, it tends to generalise across incident types, particularly in the pairing of cyberbullying with hate speech and hacking, indicating compounding risk rather than isolated vulnerability.

#### 3.3.3. Education Level and Type of TFGBV Experienced

Analysis of the dataset shows that secondary school certificate holders reported the highest number of TFGBV cases at 30.2% of the total, followed by college diploma holders (22.1%) and those with some secondary school education (20.5%). Bachelor’s degree holders accounted for 12.1% of the cases, while TVET certificate holders reported 6.7% cases. Lower prevalence was found among participants with primary school education (4.0%) and those with postgraduate (Honours, Master’s, and Doctoral) degrees (4.3%). Across all education levels, the most frequently reported TFGBV type was cyberbullying (*n* = 88), followed by hate speech (*n* = 51), hacking (*n* = 49), and threats (*n* = 28). Less frequently reported categories included online impersonation (*n* = 16), doxxing (*n* = 15), technology restriction and isolation (*n* = 16), image-based sexual abuse (*n* = 12), and cyberstalking (*n* = 7).

The mean number of cases per TFGBV type across education groups ranged from 1.00 (cyberstalking) to 12.57 (cyberbullying). The range (max–min) was highest for cyberbullying (34) and hate speech (16), suggesting substantial variation across education levels, and lowest for cyberstalking (3).

#### 3.3.4. Socio-Economic Status and Type of TFGBV Experienced

**Figure 2:**
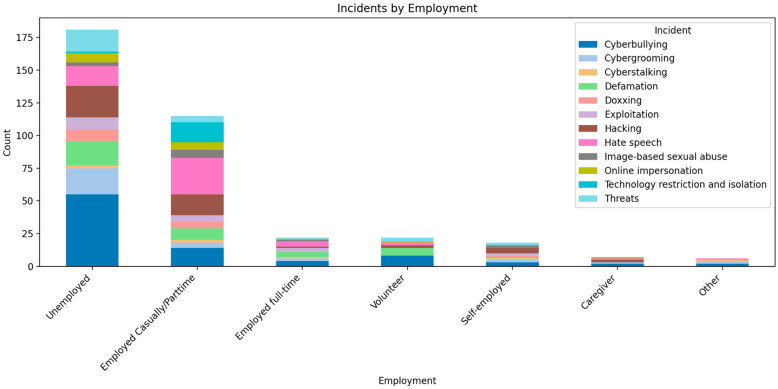
Employment and TFGBV.

Findings in this section highlight the socio-economic status data comprising the employment, income and social security grant data, in relation to the types of TFGBV experienced by participants (see Figure 2 below). Across incident types, respondents outside stable full-time employment carry a disproportionate share of cases. In the employment breakdown, cyberbullying is the top exposure across all groups, but the steepest burden sits with unemployed (*n* = 181) and precariously employed respondents (*n* = 115), who consistently register multiples of the totals observed among full-time workers (*n* = 22). In several incident categories, their counts are roughly 1.5× to 3× higher than those in stable jobs, indicating a strong positive correlation between employment instability and TFGBV. As employment security decreases, exposure to TFGBV increases.

The income distribution shows a striking concentration among the lowest income respondents. The respondents who earn less than R5000 account for the overwhelming majority of incidents across categories (*n* = 245), followed by those who earn between R6000–R20,000 (*n* = 55), those who were unsure about how much they make monthly accounted for 37 cased and followed by those who earn between R21,000 and R30,000 (*n* = 21), and only one participant who earns more than R31,000 reported a case of cybergrooming. The lowest income group commonly holds well over three-quarters of the total burden for major incident types, with the next band adding a small additional share. This pattern indicates that financial insecurity substantially elevates risk and visibility to offenders, as well as the likelihood of account compromise and reputational attacks. Additionally, social security grant recipients indicated substantially higher incident percentages than non-recipients across every major category (78% vs. 22%). Grant recipients reported significantly higher numbers for cyberbullying (*n* = 64), hate speech (*n* = 40), hacking (*n* = 41), and defamation (*n* = 30). Recipients comprise most incidents compared to non-recipients across categories, capturing well over two-thirds of exposure. This suggests that grant status is a strong proxy for economic vulnerability, tracking closely with elevated TFGBV risk.

Findings indicate that lower socio-economic status correlates with more frequent and more varied TFGBV. Employment instability, low income, and grant receipt each independently align with higher exposure. Where these factors intersect (e.g., unemployed grant recipients in the lowest income band), there is cumulative risk. The distribution of incident types adds nuance; that is, broadening of harm types at lower socio-economic status indicates not only more harassment, but more complex safety challenges, such as account security, identity and protection.

Converging evidence across employment, income, and grant status shows a consistent correlation with lower socio-economic status, including unemployment, precarious work, low income and grant receipt, which correspond with substantially higher counts of all major TFGBV incidents. The magnitude is large and consistent, often 2–5× higher for low-income groups and sometimes an order of magnitude higher in the lowest income band. The steepest gradients are observed for cyberbullying and hate speech, which together account for the majority share of incidents. However, hacking and exploitation also scale with socio-economic hardship, indicating that risk is not only social (harassment, threats) but also technical/economic.

## 4. Discussion

This study reveals a concerning prevalence of TFGBV among women with disabilities that is shaped by intersecting factors of disability, race, socio-economic status, and geographic location in South Africa. We argue that TFGBV compounds existing social inequalities. When viewed through an intersectional lens, TFGBV is not distributed evenly as factors such as disability type, race, socio-economic status, and grant dependency all shape the likelihood of various forms of harm.

This study reveals that TFGBV exposure across disability types is not monolithic. For example, individuals with physical, mobility and pain-related disabilities appear to experience higher aggregate exposure across incident types than those with sensory or intellectual disabilities. This mirrors what has been found in various studies, that women with visible (physical disabilities) are more likely to experience violence than those with invisible disabilities (Ledingham et al., 2022; Clements et al., 2024; Wolbers & Boxall, 2024). However, the types of violence which women with disabilities are subjected to vary across disability types. For example, while cyberbullying is consistently the most prevalent category, mobility and pain-related groups show elevated reported experiences of harms such as hate speech, hacking, and threats.

Furthermore, while harassment is the most commonly reported form of cyberbullying among individuals with intellectual disabilities (Clements et al., 2024), other types, such as stalking and shaming, remain common among people with intellectual disabilities. This suggests a broader surface exposure and possibly higher online participation, coupled with contentious social interactions or greater dependency on connected services.

The above-noted differences likely reflect a blend of external and structural factors, such as prior experiences with traditional bullying, increased internet use, and minority status (Waller et al., 2018). The visibility of disability and how it is perceived in online spaces can draw targeted harassment (Waller et al., 2018). Care-related and access-related dependencies may increase online footprint and the number of digital interactions, raising exposure to both opportunistic and targeted abuse (Waller et al., 2018; Huang & Wang, 2023; Wolbers & Boxall, 2024).

Findings further highlight the pronounced racial disparities, especially for cyberbullying and hate speech. These racial disparities highlight how systemic inequality and access to technology intersect with TFGBV. Communities identified as African/Black and Coloured in the data show high relative counts across multiple categories of TFGBV. This distribution across racial categories indicates well-documented patterns in the literature (Bailey & Burkell, 2021; Bansal et al., 2023; Hameed et al., 2025). Women with disabilities who also belong to racial, ethnic, or other minority groups are at increased risk of TFGBV due to intersecting oppressions such as racism, ableism, and sexism (Hameed et al., 2025). Racially marginalised communities face not merely higher counts, but cumulative harms that can silence participation, shape mental health outcomes, and curtail professional opportunities (Bansal et al., 2023).

This study also highlights that low socio-economic status, including low education levels, low income and dependency on social security grants, is strongly tied to risk of TFGBV. Lower formal education levels correlate with higher exposure for several TFGBV categories, while higher education levels show lower counts and a narrower spread across incident types. This is consistent with the broader literature that indicates that higher educational attainment is a strong protective factor (Arenas et al., 2023). Women with disabilities who have a university degree or higher education are less likely to experience abuse, including psychological and sexual abuse, compared to those with little or no education (Kwagala & Galande, 2022; Arenas et al., 2023). Lower-income brackets exhibit markedly higher totals across TFGBV incident types, and grant recipients show substantially greater exposure than non-recipients.

Several mechanisms may be at play. The first mechanism is that access and gatekeeping, where lower-income users may rely on lower-cost devices, shared connections, or public Wi-Fi, increase susceptibility to hacking, doxxing, and impersonation through weaker security and exposure to untrusted networks (Jain & Agrawal, 2020; Bansal et al., 2023). The second mechanism is that fraud and exploitation targeting is likely to increase—where the lack of economic resources is a known risk factor for grooming, exploitation, and scam-adjacent harms that escalate into TFGBV, particularly where gendered dynamics intersect with caregiver responsibilities and financial stress (Henry & Flynn, 2019). Thirdly, platform vulnerability exists, where lower digital literacy and fewer resources for premium moderation/verification features can exacerbate exposure and reduce recovery (Jain & Agrawal, 2020). Grant recipients, a proxy for socio-economic vulnerability and dependency on state support, face elevated risk across almost every category, suggesting a broader vulnerability pattern. Paradoxically, receiving public economic aid is associated with a higher risk of abuse. Consistent with the literature, women with disabilities who rely on financial assistance are more likely to experience domestic and sexual violence than those who do not receive such aid (Arenas et al., 2023).

This study shows that TFGBV is deeply intersectional. Individuals navigating multiple marginalised identities, especially where disability, race, and low income intersect, face broader and more frequent harm. Cyberbullying dominance is only the most visible part of a multiplex risk environment that includes hate speech, threats, doxxing, impersonation, and hacking. The patterns that emerge are both intuitively coherent and actionable, pointing to urgent needs for targeted interventions, platform accountability, and policy safeguards calibrated to specific vulnerabilities. The path forward is both technical and social. We recommend that safety-by-design must meet community-informed moderation and targeted policy support, with specific attention to those most impacted. By aligning platform design, public policy, and community practice to the patterns revealed herein, exposure can be reduced.

### Limitations

While this study offers important insights into the intersectional experiences of TFGBV among women with disabilities, several limitations must be acknowledged.

First, although this is a national survey, there were challenges with recruiting participants from across the country. The researchers are situated in the Western Cape province and conducted information-sharing sessions as an additional means for recruitment, hence, the larger number of participants from this province. Moreover, issues around digital connectivity and affordability may be unevenly distributed across the South African context, which need to be taken into account in future TFGBV research associated with this particular population group.

Secondly, the use of an online survey, though appropriate for the topic, may have excluded women with disabilities who lack consistent access to digital technologies, the internet, or accessible devices. This may have led to an underrepresentation of certain groups, particularly those living in rural areas, older women, and those with higher support needs or lower digital literacy.

Thirdly, the self-report nature of the survey introduces potential biases, including underreporting due to stigma, fear of retaliation, or uncertainty about whether an experience qualifies as violence. Conversely, some participants with greater digital awareness may have been more likely to recognise and report TFGBV experiences.

Fourthly, while the study applied an intersectional lens, the survey’s structure limited the depth of qualitative insights into the lived experiences behind the statistical patterns. Additionally, the survey was also limited on the gendered analysis as it only asked participants to indicate their sexual orientation, not their gender identity. Particularly of note is that half of the participants reported their identity as lesbian (49.5%). This number is disproportionately large compared to the South African demographic, and very likely due to a misattribution between gender and sexuality. Upon further exploration of the literature around the potential pattern of lesbian-identifying women with disabilities, we discovered that the cause may be due to the researchers’ own use of Western-centric or colonial language. Particularly, there is a common understanding that discourse around sex and gender may not translate well while navigating a multilingual, post-colonial context (Lynch et al., 2021). This is a pervasive issue when attempting to directly translate a survey designed from a majorly Western-centric or Anglo-centric lens into a South African one. Future research in the area needs to explicitly identify trans women as a participant group and to determine the appropriate definitions and terminology around sexualities and gender identities. Moreover, co-design processes must explicitly prioritise representative gender-diverse and LGBTQI+ partners, and native speakers of all translated languages, within the co-design process to ensure that both content, structure and format are inclusive of trans women and other diverse gender identities with disabilities, and easily identifiable for them. Lastly, the sample size, though national in scope, was relatively small and may not fully capture the heterogeneity of experiences among women with different types of disabilities across South Africa. The uneven geographic distribution of respondents constrains the interpretation of the spatial analysis. Spatial patterns observed in the data may reflect differential recruitment reach, internet accessibility, and organisational presence rather than true geographic variation in TFGBV experiences. Consequently, spatial findings should be interpreted as exploratory and descriptive, rather than as precise estimates of regional prevalence or risk. These limitations highlight the need for future research employing stratified or regionally representative sampling approaches and enhanced outreach strategies to strengthen spatial inference and generalizability. Despite these limitations, the findings provide a valuable baseline for understanding TFGBV among women with disabilities and highlight the urgency of further research and action in this area.

## 5. Conclusions

This study demonstrates that TFGBV against women with disabilities in LMICs is shaped by intersecting inequalities that amplify both risk and exclusion. Disability, gender, race and socio-economic status combine to produce unique vulnerabilities, limiting access to digital safety, justice, and support. The findings emphasise that interventions must go beyond individual responses to address the structural, institutional, and economic factors that underpin TFGBV. An intersectional, power-conscious framework is critical to developing equitable, inclusive strategies that centre the voices and experiences of women with disabilities. Such an approach enables not only the prevention of online violence but also the dismantling of broader systems of inequality that perpetuate exclusion, advancing both gender justice and disability rights. Future policy interventions must centre on disabled women’s experiences and address the structural inequalities that make them disproportionately vulnerable online.

## Figures and Tables

**Table 1:** TFGBV incidents across racial groups.

Category	Mean	Median	Mode	Range	Total
African/Black	20.25	13.5	12	50	243
Coloured	7.666667	6	6	26	92
White	2.333333	2.5	1	4	28
Indian	0.666667	0.5	0	2	8

## Data Availability

In line with institutional ethics approval for this research, no data is publicly available.

## Note

1 In the South African context, this is an apartheid racial category which referred to people of mixed blood/race. They mostly speak Afrikaans. In this study, we refer to Coloured as a cultural identity, not skin colour (Erasmus, 2001).

## Abbreviations

The following abbreviations are used in this manuscript:

GBV	Gender-Based Violence
ICTs	Information and Communication Technologies
LMIC	Low- and Middle-Income Countries
TFGBV	Technology-Facilitated Gender-Based Violence

## Appendix A. Survey Terminology and Definitions

**Types of Disabilities**

**Hearing Disability**: You have a hard time hearing clearly, even when you use hearing aids or cochlear implants. This includes being a Deaf or hard of hearing person, or having tinnitus (hearing constant ringing or buzzing sounds).

**Vision Disability**: You have a hard time seeing clearly, even when you use glasses or contact lenses. This includes being a blind or legally blind person.

**Mobility or Dexterity Disability**: You have a hard time moving comfortably. This might look like having a hard time…

- Moving around, even when using a support, like a cane. Walking on a flat surface for 15 min. Walking up or down a flight of stairs (about 12 steps).
- Bending over and picking up an object from the floor.
- Reaching in any direction, for example, above your head.
- Picking up small objects with your fingers, like a pencil or scissors.

**Pain-related Disability**: You have long-term pain which impacts your daily life. This may include…

- Pain caused by another condition which lasts for 6 months or more.
- Pain that is always there.
- Ongoing pain that happens from time to time.

**Intellectual or Learning**: You have a condition that makes it hard for you to learn. This may include conditions like dyslexia, attention deficit hyperactivity disorder (ADHD), dyscalculia (a learning disorder that makes it hard to understand numbers and math).

**Developmental**: You have a disability which developed as a child. This might include Down syndrome, fetal alcohol syndrome, spina bifida or being on the autism spectrum.

**Memory**: You may have consistent memory problems or periods of confusion. These problems may be caused by diseases such as Alzheimer’s and other kinds of dementia, or may be the result of a brain injury, such as a concussion.

**Psycho-social Disability**: You have a mental health-related condition that has lasted or will probably last for six months or longer. These may include mood conditions such as anxiety, depression, and bipolar disorder, substance addiction or abuse, and eating disorders such as anorexia and bulimia nervosa.

## Appendix B. Types of Technology-Facilitated Gender-Based Violence (TFGBV)

**Cyberbullying:** Cyberbullying is when someone repeatedly and intentionally tries to hurt another person online. This can be physical, social, or emotional harm. It often involves one person or a group using their power to target someone who feels that they are vulnerable, or unable to stop the bullying.

**Cybergrooming:** Cybergrooming is when someone uses the internet to build trust with a person to take advantage of them sexually or financially. The person might act like a friend or pretend to care about them to make them feel safe. Over time, they use this trust to make the person agree to abusive behaviour or to manipulate them into giving money or access to financial resources.

**Defamation:** Cyber-defamation is when someone spreads lies about another person online to hurt them and/or their reputation.

**Exploitation:** Cyber-exploitation is when someone uses the internet to abuse or force another person to do something. The person doing this gets some kind of benefit, like money, social status, or political power.

**Hacking:** Hacking is the activity is when someone uses digital technology to access information stored on someone else’s digital device without permission, or to spread a computer/mobile virus.

**Hate speech:** Hate speech is when someone uses words, images, or actions to insult, harass, threaten, or promote violence against a person or group because of things like their gender, disability, sexual orientation, or race. For example, making fun of women, encouraging violence against them, or treating their struggles as unimportant are all forms of hate speech.

**Image-based sexual abuse:** Image-based sexual abuse involves taking or sharing intimate photos or videos without permission or threatening to share them. It also includes things like:

- ‘Sextortion’: when someone blackmails another person by threatening to release explicit or private images of them;
- ‘Revenge porn’: when intimate content is shared without a person’s consent;
- Sharing child sexual exploitation materials with people who do not know about it.

**Doxxing:** Doxxing is when someone finds and shares private information about another person on the internet without their permission. This can include things like their name, address, and other personal details. Sometimes this can lead to the target person being harmed in some way.

**Online impersonation:** Online impersonation is when someone makes a fake social media account using a target person’s name or photo, and posts hurtful or offensive things. This is usually done to ruin the person’s reputation or cause more trouble. Sometimes, the person doing this will pretend to be someone who the target knows, to harm them.

**Surveillance and cyberstalking:** This can include actions (such as texts, direct messages, phone calls, etc.) which involve repeated or unwanted attention, harassment, or contact, causing reasonable fear for a target.

**Threats:** Cyber-based threats are online messages or actions meant to scare, harm, or suggest that something unpleasant or violent will happen to someone. This can include things like sending scary messages, threatening to hurt someone, or making them feel unsafe on the internet.

**Technology restriction and isolation:** Technology restriction and isolation happens when someone stops another person from using phones, computers, the internet or other forms of technology. They may do this to cut them off from talking to friends, family, or getting help (this is called isolating them). They may also do this to control what the person does, like who they talk to or what they see online. This makes it easier for the abuser to harm or neglect them.
